# Supplementary material for: Medical students’ and educators’ opinions of teleconsultation in practice and undergraduate education: A UK-based mixed-methods study
Source: PLoS One. 2025 Mar 6;20(3):e0302088. doi: 10.1371/journal.pone.0302088 (PMC11884699; doi:10.1371/journal.pone.0302088)
Supplement: S1 File — This questionnaire was distributed to UK undergraduate medical students and medical educators. (DOCX) [file pone.0302088.s001.docx]

# S1 File

Start of Block: Information

You are invited to participate in a research study titled '*Acceptance and perception of TC by medical undergraduate students and faculty*'. This study is being conducted as part of a PhD Thesis by Lisa Wetzlmair from the School of Medicine at the University of St Andrews. What is the study about?

**The aim of this research is to gain understanding of** **the acceptance and opinion of TC education in the UK.**

Why have I been invited to take part? You have been invited to take part in this study, because you are an undergraduate medical student, currently studying in the UK, a member of faculty at a Medical School in the UK, or involved in the education of medical students. What would I be required to do?

Complete an online questionnaire which contains 30 questions that should take around **15 minutes to complete**. After the survey, you will also have to option to participate in an interview via telephone or video-call that will take around 30 minutes. You do not have to take part in the interview but if you do wish to be part, we will ask you to provide your contact details. We will use this information only to contact you for setting up the interview. 

If you are interested in taking part, please download a copy of the participant information sheet [here](https://standrews.eu.qualtrics.com/CP/File.php?F=F_0TyZsTrnWQi8uBU). Retain this for your records before starting the questionnaire. If you have any questions, please email telecom@st-andrews.ac.uk Your participation is entirely voluntary, and you can withdraw at any time. You are free to omit any question.

- Yes, I confirm that I am willing to take part in this research.
- No, I do not confirm that I am willing to take part in this research.

End of Block: Information

Start of Block: General information

Q1: The next questions are to gather some demographic information about you. **What role best describes your current status in medical education?**

- Student
- Medical Educator within an academic setting
- Medical Educator working within both an academic setting and a healthcare setting
- Medical Educator working within the healthcare setting
- Other, please specify ________________________________________________

Q2: What is your gender?

- Male
- Female
- Non-binary / third gender
- Prefer not to say

Display This Question:

If Q1: The next questions are to gather some demographic information about you. What role best desc... = Student

Q3a: Which UK Medical School do you currently attend?

▼ University of Aberdeen School of Medicine and Dentistry (2) ... other (46)

Display This Question:

If Q1: The next questions are to gather some demographic information about you. What role best desc... = Student

Q3b: What year are you in?

▼ Year 1 (1) ... Intercalating year (9)

Display This Question:

If Q1: The next questions are to gather some demographic information about you. What role best desc... = Medical Educator within an academic setting

Or Q1: The next questions are to gather some demographic information about you. What role best desc... = Medical Educator working within both an academic setting and a healthcare setting

Or Q1: The next questions are to gather some demographic information about you. What role best desc... = Medical Educator working within the healthcare setting

Or Q1: The next questions are to gather some demographic information about you. What role best desc... = Other, please specify

Q3: Which UK Medical School are you affiliated with? Choose all options, that apply for you at the moment.

- University of Aberdeen School of Medicine and Dentistry
- Anglia Ruskin University School of Medicine
- Aston University Medical School
- Barts and The London School of Medicine and Dentistry
- University of Birmingham College of Medical and Dental Sciences
- Brighton and Sussex Medical School
- University of Bristol Medical School
- University of Buckingham Medical School
- University of Cambridge School of Clinical Medicine
- Cardiff University School of Medicine
- University of Dundee School of Medicine
- Edge Hill University Medical School
- The University of Edinburgh Medical School
- University of Exeter Medical School
- University of Glasgow School of Medicine
- Hull York Medical School
- Imperial College London Faculty of Medicine
- Keele University School of Medicine
- Kent and Medway Medical School
- King's College London GKT School of Medical Education
- Lancaster University Medical School
- University of Leeds School of Medicine
- University of Leicester Medical School
- University of Liverpool School of Medicine
- London School of Hygiene & Tropical Medicine
- University of Manchester Medical School
- Newcastle University School of Medical Education
- Norwich Medical School
- University of Nottingham School of Medicine
- University of Nottingham - Lincoln Medical School
- University of Oxford Medical Sciences Division
- Plymouth University Peninsula Schools of Medicine and Dentistry
- Queen's University Belfast School of Medicine
- University of Sheffield Medical School
- University of Southampton School of Medicine
- University of St Andrews School of Medicine
- St George's, University of London
- University of Sunderland School of Medicine
- Swansea University Medical School
- University of Central Lancashire School of Medicine
- University College London Medical School
- University of Warwick Medical School
- Ulster University
- other, please specify ____________________________________________

Q4: What is your highest level of education completed to date?

- High School
- College / Further Education
- Bachelor's Degree
- Master's Degree
- PhD
- MD
- other, please specify ________________________________________________

Display This Question:

If Q1: The next questions are to gather some demographic information about you. What role best desc... = Medical Educator working within both an academic setting and a healthcare setting

How many hours per week do you work for a university? : _______

How many hours per week do you work for a healthcare setting? : _______

Total : ________

End of Block: General information

Start of Block: Experience with teleconsultation

For this survey, **teleconsultation** is defined as "**synchronous or asynchronous consultation using information and communication technology to omit geographical and functional distance. Its goals are for diagnostics or treatment between two or more geographically separated health providers (for example physicians or nurses) or between health providers and patients"**(Deldar et al., 2016).

Examples of teleconsultation are: Practitioner (video-)calls patient for a regular follow-up. Practitioner (video-)calls patient to discuss blood results.

Patient emails practitioner with a question about new medication.

Patient (video-)calls practitioner with an acute medical problem.

**Teleconsultation technologies** are all technologies that are associated with teleconsultation (Maarop et al., 2011). Examples of teleconsultation technologies are:

Information Communication Technology (e.g. telephone, smartphone, email, chat) Videoconferencing technology (e.g. video equipment, video camera, microphone)

Display This Question:

If Q1: The next questions are to gather some demographic information about you. What role best desc... = Medical Educator within an academic setting

Or Q1: The next questions are to gather some demographic information about you. What role best desc... = Medical Educator working within both an academic setting and a healthcare setting

Or Q1: The next questions are to gather some demographic information about you. What role best desc... = Medical Educator working within the healthcare setting

Or Q1: The next questions are to gather some demographic information about you. What role best desc... = Other, please specify

5a: Have you **offered** teleconsultation services (i.e. consultations via telephone or video-call) in **healthcare**?

- Yes, before COVID-19
- Yes, during COVID-19
- Yes, before and during COVID-19
- No

Display This Question:

If Q1: The next questions are to gather some demographic information about you. What role best desc... = Medical Educator within an academic setting

Or Q1: The next questions are to gather some demographic information about you. What role best desc... = Medical Educator working within both an academic setting and a healthcare setting

Or Q1: The next questions are to gather some demographic information about you. What role best desc... = Medical Educator working within the healthcare setting

Or Q1: The next questions are to gather some demographic information about you. What role best desc... = Other, please specify

Q5b: Have you **taught** students teleconsultation techniques (i.e. consultations via telephone or video-call) **at university**?

- Yes, before COVID-19
- Yes, during COVID-19
- Yes, before and during COVID-19
- No

Display This Question:

If Q1: The next questions are to gather some demographic information about you. What role best desc... = Student

Q5a: Have you **learned** about teleconsultation techniques (i.e. consultations via telephone or video-call) in your **clinical placements**?

- Yes, before COVID-19
- Yes, during COVID-19
- Yes, before and during COVID-19
- No

Display This Question:

If Q1: The next questions are to gather some demographic information about you. What role best desc... = Student

Q5b: Have you **learned** about teleconsultation techniques (i.e. consultations via telephone or video-call) in a **university setting**?

- Yes, before COVID-19
- Yes, during COVID-19
- Yes, before and during COVID-19
- No

End of Block: Experience with teleconsultation

Start of Block: Technology Acceptance of Teleconsultation

Display This Question:

If Q1: The next questions are to gather some demographic information about you. What role best desc... = Student

| 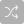 |
| --- |

Q6: The next part of the questionnaire will ask you about your attitudes towards teleconsultation.

When completing the following questions, consider your future practice and how you think you will use teleconsultation.

Consider, which response best reflects your opinion as a future practitioner. Should you find some questions repetitive, please answer each question on its own merit.

|  | Strongly agree (1) | Somewhat agree (2) | Neither agree nor disagree (3) | Somewhat disagree (4) | Strongly disagree (5) |
| --- | --- | --- | --- | --- | --- |
| I feel prepared for working in a digitized healthcare system. |  |  |  |  |  |
| Offering teleconsultation services would enable me to complete patient care more quickly. |  |  |  |  |  |
| Offering teleconsultation services would increase my productivity in patient care. |  |  |  |  |  |
| Offering teleconsultation services would enhance my service effectiveness. |  |  |  |  |  |
| Offering teleconsultation services would make my patient care and management easier. |  |  |  |  |  |
| Teleconsultation services would improve my care and management. |  |  |  |  |  |
| Offering teleconsultation services would be useful for my patient care and management. |  |  |  |  |  |
| Learning to offer teleconsultation services would be hard for me. |  |  |  |  |  |
| I would find it easy to get teleconsultation technology to do what I need to do in my patient care and management. |  |  |  |  |  |
| Interacting with teleconsultation technology would be clear and understandable for me. |  |  |  |  |  |
| I would find teleconsultation technology to be flexible to interact with. |  |  |  |  |  |
| I would find it easy to become skillful at offering teleconsultation services. |  |  |  |  |  |
| I would find teleconsultation technology easy to use. |  |  |  |  |  |
| Offering teleconsultation services in patient care and management would be a good idea. |  |  |  |  |  |
| Offering teleconsultation services in patient care and management would be unpleasant. |  |  |  |  |  |
| Offering teleconsultation services would be beneficial to my patient care and management. |  |  |  |  |  |
| I would like the idea of offering teleconsultation services. |  |  |  |  |  |
| Overall, I would enjoy using teleconsultation services. |  |  |  |  |  |
| I intend to offer teleconsultation services for patient care and management as often as needed. |  |  |  |  |  |
| Whenever possible, I do not intend to offer teleconsultation services for patient care and management. |  |  |  |  |  |
| I would offer teleconsultation services in my patient care and management frequently. |  |  |  |  |  |
| I intend to offer teleconsultation services in my patient care and management when it becomes available in my department or hospital. |  |  |  |  |  |

Display This Question:

If Q1: The next questions are to gather some demographic information about you. What role best desc... = Medical Educator working within both an academic setting and a healthcare setting

Or Q1: The next questions are to gather some demographic information about you.  What role best desc... = Medical Educator working within the healthcare setting

| 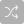 |
| --- |

Q6: The next part of the questionnaire will ask you about your attitudes towards teleconsultation. Consider, which response best reflects your opinion at the moment.
Should you find some questions repetitive, please answer each question on its own merit.

|  | Strongly agree (1) | Somewhat agree (2) | Neither agree nor disagree (3) | Somewhat disagree (4) | Strongly disagree (5) |
| --- | --- | --- | --- | --- | --- |
| I feel prepared for working in a digitized healthcare system. |  |  |  |  |  |
| Offering teleconsultation services enables me to complete patient care more quickly. |  |  |  |  |  |
| Offering teleconsultation services increases my productivity in patient care. |  |  |  |  |  |
| Offering teleconsultation services enhances my service effectiveness. |  |  |  |  |  |
| Offering teleconsultation services makes my patient care and management easier. |  |  |  |  |  |
| Teleconsultation services improve my care and management. |  |  |  |  |  |
| Offering teleconsultation services is useful for my patient care and management. |  |  |  |  |  |
| Learning to offer teleconsultation services is hard for me. |  |  |  |  |  |

| I find it easy to get teleconsultation technology to do what I need to do in my patient care and management. |  |  |  |  |  |
| --- | --- | --- | --- | --- | --- |
| Interacting with teleconsultation technology is clear and understandable for me. |  |  |  |  |  |
| I find teleconsultation technology to be flexible to interact with. |  |  |  |  |  |
| I find it easy to become skillful at offering teleconsultation services. |  |  |  |  |  |
| I find teleconsultation technology easy to use. |  |  |  |  |  |
| Offering teleconsultation services in patient care and management is a good idea. |  |  |  |  |  |
| Offering teleconsultation services in patient care and management is unpleasant. |  |  |  |  |  |
| Offering teleconsultation services is beneficial to my patient care and management. |  |  |  |  |  |
| I like the idea of offering teleconsultation services. |  |  |  |  |  |
| Overall, I enjoy offering teleconsultation services. |  |  |  |  |  |
| I intend to offer teleconsultation services for patient care and management as often as needed. |  |  |  |  |  |
| Whenever possible, I do not intend to offer teleconsultation services for patient care and management. |  |  |  |  |  |
| I would offer teleconsultation services in my patient care and management frequently. |  |  |  |  |  |
| I intend to offer teleconsultation services in my patient care and management when it becomes available in my department or hospital. |  |  |  |  |  |

Display This Question:

If Q1: The next questions are to gather some demographic information about you. What role best desc... = Medical Educator within an academic setting

Or Q1: The next questions are to gather some demographic information about you. What role best desc... = Other, please specify

| 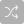 |
| --- |

Q6: The next part of the questionnaire will ask you about your attitudes towards teleconsultation. When completing the following questions, think of former patient encounters or imagine teleconsultation being used in patient care and management.
 Should you find some questions repetitive, please answer each question on its own merit. *If any of these questions do not apply to you, please feel free to click "not applicable".*

|  | Strongly agree (30) | Somewhat agree (31) | Neither agree nor disagree (32) | Somewhat disagree (34) | Strongly disagree (37) | Not applicable (38) |
| --- | --- | --- | --- | --- | --- | --- |
| I feel prepared for working in a digitized healthcare system. |  |  |  |  |  |  |
| Offering teleconsultation services enables me to complete patient care more quickly. |  |  |  |  |  |  |
| Offering teleconsultation services increases my productivity in patient care. |  |  |  |  |  |  |
| Offering teleconsultation services enhances my service effectiveness. |  |  |  |  |  |  |

| Offering teleconsultation services makes my patient care and management easier. |  |  |  |  |  |  |
| --- | --- | --- | --- | --- | --- | --- |
| Teleconsultation services improve my care and management. |  |  |  |  |  |  |
| Offering teleconsultation services is useful for my patient care and management. |  |  |  |  |  |  |
| Learning to offer teleconsultation services is hard for me. |  |  |  |  |  |  |
| I find it easy to get teleconsultation technology to do what I need to do in my patient care and management. |  |  |  |  |  |  |
| Interacting with teleconsultation technology is clear and understandable for me. |  |  |  |  |  |  |
| I find teleconsultation technology to be flexible to interact with. |  |  |  |  |  |  |
| I find it easy to become skillful at offering teleconsultation services. |  |  |  |  |  |  |
| I find teleconsultation technology easy to use. |  |  |  |  |  |  |
| Offering teleconsultation services in patient care and management is a good idea. |  |  |  |  |  |  |
| Offering teleconsultation services in patient care and management is unpleasant. |  |  |  |  |  |  |
| Offering teleconsultation services is beneficial to my patient care and management. |  |  |  |  |  |  |
| I like the idea of offering teleconsultation services. |  |  |  |  |  |  |
| Overall, I enjoy offering teleconsultation services. |  |  |  |  |  |  |
| I intend to offer teleconsultation services for patient care and management as often as needed. |  |  |  |  |  |  |
| Whenever possible, I do not intend to offer teleconsultation services for patient care and management. |  |  |  |  |  |  |
| I would offer teleconsultation services in my patient care and management frequently. |  |  |  |  |  |  |
| I intend to offer teleconsultation services in my patient care and management when it becomes available in my department or hospital. |  |  |  |  |  |  |

End of Block: Technology Acceptance of Teleconsultation

Start of Block: Difficulties in use and self-evaluation

Display This Question:

If Q1: The next questions are to gather some demographic information about you. What role best desc... = Student

And Q1: The next questions are to gather some demographic information about you. What role best desc... = Medical Educator working within both an academic setting and a healthcare setting

And Q1: The next questions are to gather some demographic information about you. What role best desc... = Medical Educator working within the healthcare setting

And Q1: The next questions are to gather some demographic information about you. What role best desc... = Other, please specify

Q7a: Do you intend to use teleconsultation services in your future practice?

- Yes, I will continue to use existing service and technologies. (1)
- Yes, but with improvements. (2)
- No. (3)

Display This Question:

If Q1: The next questions are to gather some demographic information about you. What role best desc... = Medical Educator within an academic setting

And Q1: The next questions are to gather some demographic information about you. What role best desc... = Medical Educator working within both an academic setting and a healthcare setting

Q7b: Do you intend to teach teleconsultation services in the future?

- Yes, I will continue to use existing service and technologies. (1)
- Yes, but with improvements. (2)
- No. (3)

Display This Question:

If Q5a: Have you learned about teleconsultation techniques (i.e. consultations via telephone or vide... = Yes, before COVID-19

Or Or Q5a: Have you learned about teleconsultation techniques (i.e. consultations via telephone or video-call) in your clinical placements? q://QID37/SelectedChoicesCount Is Not Empty

Or Q5a: Have you learned about teleconsultation techniques (i.e. consultations via telephone or vide... = Yes, during COVID-19

Or Q5a: Have you learned about teleconsultation techniques (i.e. consultations via telephone or vide... = Yes, before and during COVID-19

Or If

Q5b: Have you learned about teleconsultation techniques (i.e. consultations via telephone or vide... = Yes, before COVID-19

Or Or Q5b: Have you learned about teleconsultation techniques (i.e. consultations via telephone or video-call) in a university setting? q://QID65/SelectedChoicesCount Is Not Empty

Or Q5b: Have you learned about teleconsultation techniques (i.e. consultations via telephone or vide... = Yes, during COVID-19

Or Q5b: Have you learned about teleconsultation techniques (i.e. consultations via telephone or vide... = Yes, before and during COVID-19

Q8a: Consider difficulties you have experienced with teleconsultation services either in **healthcare or University settings.**

|  | No, never (1) | Yes, sometimes (2) | Yes, often (3) |
| --- | --- | --- | --- |
| Have you experienced technical difficulties that may have affected the quality of care provided by teleconsultation services? |  |  |  |
| Have you experienced organisational or other difficulties that may have affected the quality of care provided by teleconsultation services? |  |  |  |

Display This Question:

If Q5a: Have you offered teleconsultation services (i.e. consultations via telephone or video-call)... = Yes, before COVID-19

Or Q5a: Have you offered teleconsultation services (i.e. consultations via telephone or video-call)... = Yes, during COVID-19

Or Q5a: Have you offered teleconsultation services (i.e. consultations via telephone or video-call)... = Yes, before and during COVID-19

Or Or Q5a: Have you offered teleconsultation services (i.e. consultations via telephone or video-call)... q://QID35/SelectedChoicesCount Is Not Empty

Q8b: Consider difficulties you have experienced with teleconsultation services in your **current practise.**

|  | No, never (1) | Yes, sometimes (2) | Yes, often (3) |
| --- | --- | --- | --- |
| Have you experienced technical difficulties that may have affected the quality of care provided by teleconsultation services? |  |  |  |
| Have you experienced organisational or other difficulties that may have affected the quality of care provided by teleconsultation services? |  |  |  |

Display This Question:

If Q5b: Have you taught students teleconsultation techniques (i.e. consultations via telephone or vi... = Yes, before COVID-19

Or Q5b: Have you taught students teleconsultation techniques (i.e. consultations via telephone or vi... = Yes, during COVID-19

Or Q5b: Have you taught students teleconsultation techniques (i.e. consultations via telephone or vi... = Yes, before and during COVID-19

Or Or Q5b: Have you taught students teleconsultation techniques (i.e. consultations via telephone or vi... q://QID36/SelectedChoicesCount Is Not Empty

Q8c: Consider difficulties you have experienced with teleconsultation services in your **teaching.**

|  | No, never (1) | Yes, sometimes (2) | Yes, often (3) |
| --- | --- | --- | --- |
| Have you experienced technical difficulties that may have affected the quality of your teaching using teleconsultation technology? |  |  |  |
| Have you experienced organisational or other difficulties that may have affected the quality of your teaching using teleconsultation technology? |  |  |  |

Q9: I evaluate my teleconsultation skills (e.g. remote patient history taking, remote patient physical examination) as ...

- very good (1)
- good (2)
- acceptable (3)
- poor (4)
- very poor (5)

End of Block: Difficulties in use and self-evaluation

Start of Block: Former training

Q10: **How** do students learn about teleconsultation services at your University?
 (Mark all options that apply)

- Lecture
- Clinical placements
- Simulated environment (e.g. clinical skills)
- Self-directed
- Small group tutorials (e.g. problem-based learning, case-based learning)
- other (please specify) __________________________________________
- Don't know

Q11: **What** do students learn about teleconsultation services at your University? (Mark all options that apply)

- Usage of TC technologies
- Ethical discussions
- Computer Science (e.g. Data Science, programming languages)
- Start-up possibilities
- Research opportunities (e.g. usage of big data)
- No teaching at all
- other (please specify) ___________________________________________
- Don't know

Q12: When do students learn about teleconsultation services at your University?

- First half of the programme
- Second half of the programme
- Throughout the whole programme
- Don't know

Q13: Is the teleconsultation education at your University mandatory or voluntary?

- Mandatory
- Voluntary
- A combination of both
- Don't know

Q14: I would like TC to be more implemented in the medical curriculum.

- Strongly agree
- Agree
- Neither agree nor disagree
- Disagree
- Strongly disagree

End of Block: Former training

Start of Block: Advantages / Disadvantages

Q15a: Drag and drop: Please rank the following **advantages** of teleconsultations for **health workers.** Start with the most important advantage in your opinion.

_ Teleconsultations save time.

_ Teleconsultations save costs.

_ Teleconsultations reduce the transmission of COVID-19.

_ Teleconsultations are ideal for patients with long term conditions.

_ Teleconsultations improve access to healthcare for remote populations.

_ Collaboration and communication with other health workers is easier via videoconsultation.

_ Teleconsultations decrease the amount of unnecessary medical procedures.

Q15b: Drag and drop: Please rank the following **disadvantages** of teleconsultations for **health workers.**Start with the most important disadvantage in your opinion.

_ Technical problems can interrupt or delay videoconsultations.

_ Teleconsultations are incompatible with physical examinations.

_ Not all health workers have the technical resources for teleconsultations (e.g. video equipment).

_ Not all health workers are trained in conducting teleconsultations.

_ Teleconsultations increase concerns about confidentiality and data security.

_ Delivering difficult or upsetting news during teleconsultations is harder.

Q16a: Drag and drop: Please rank the following **advantages**of teleconsultations for **patients.**Start with the most important advantage in your opinion.

_ Teleconsultations save time (e.g. for travelling, waiting in the surgery, etc.).

_ Teleconsultation save costs (e.g. for travelling)

_ Teleconsultations reduce the transmission of COVID-19.

_ Teleconsultations are ideal for patients with long term conditions.

_ Health services can be offered to underserved areas.

_ Patients can have teleconsultations with various health workers at the same time.

_ Teleconsultations can decrease the amount of unnecessary medical procedures.

Q16b: Drag and drop: Please rank the following **disadvantages**of teleconsultations for **patients.**Start with the most important disadvantage in your opinion.

_ Technical problems can interrupt or delay teleconsultations.

_ Limited non-verbal cues can cause communication problems and difficulties.

_ Not all patients have access to the required technology (e.g. video equipment).

_ Not all patients have knowledge and competencies needed for teleconsultations.

_ Teleconsultations increase concerns about confidentiality and data security.

_ Delivering difficult or upsetting news during teleconsultations is harder.

Q17: Can you think of any other challenges, stressors, disadvantages, benefits, opportunities, or advantages of teleconsultations, which were not mentioned above?

________________________________________________________________

End of Block: Advantages / Disadvantages

Start of Block: End of survey / Interview

Thank you for participating in this study. Your information is very valuable. By clicking the ‘Submit’ button below, you are consenting to participate in this study, as it is described in the participant information sheet, which you can download [here](https://standrews.eu.qualtrics.com/CP/File.php?F=F_0TyZsTrnWQi8uBU). If you did not yet download and keep a copy of this document for your records, we recommend you do that now.

- Submit

Skip To: End of Survey If Thank you for participating in this study. Your information is very valuable. By clicking the ‘... = Submit

End of Block: End of survey / Interview
