# Supplementary material for: Medical students’ and educators’ opinions of teleconsultation in practice and undergraduate education: A UK-based mixed-methods study
Source: PLoS One. 2025 Mar 6;20(3):e0302088. doi: 10.1371/journal.pone.0302088 (PMC11884699; doi:10.1371/journal.pone.0302088)
Supplement: S2 Table — Table showing the references that informed the ranking items used in the questionnaire. (DOCX) [file pone.0302088.s002.docx]

# S2 Table

| **Advantages** | **Supporting references** |
| --- | --- |
| Teleconsultations save time | Almathami, H. K. Y., Win, K. T., & Vlahu-Gjorgievska, E. (2020). Barriers and facilitators that influence telemedicine-based, real-time, online consultation at patients' homes: systematic literature review. *J Med Internet Res*, *22*(2), e16407. <https://doi.org/10.2196/16407>  Ignatowicz, A., Atherton, H., Bernstein, C. J., Bryce, C., Court, R., Sturt, J., & Griffiths, F. (2019). Internet videoconferencing for patient-clinician consultations in long-term conditions: A review of reviews and applications in line with guidelines and recommendations. *Digit Health*, *5*, 2055207619845831. <https://doi.org/10.1177/2055207619845831>  Mallow, J. A., Petitte, T., Narsavage, G., Barnes, E., Theeke, E., Mallow, B. K., & Theeke, L. A. (2016). The Use of Video Conferencing for Persons with Chronic Conditions: A Systematic Review. *Ehealth Telecommun Syst Netw*, *5*(2), 39-56. <https://doi.org/10.4236/etsn.2016.52005> |
| Teleconsultations save costs | Mallow, J. A., Petitte, T., Narsavage, G., Barnes, E., Theeke, E., Mallow, B. K., & Theeke, L. A. (2016). The Use of Video Conferencing for Persons with Chronic Conditions: A Systematic Review. *Ehealth Telecommun Syst Netw*, *5*(2), 39-56. <https://doi.org/10.4236/etsn.2016.52005>  Ruf, B., Jenkinson, P., Armour, D., Fraser, M., & Watson, A. J. (2020). Videoconference clinics improve efficiency of inflammatory bowel disease care in a remote and rural setting. *J Telemed Telecare*, *26*(9), 545-551. <https://doi.org/10.1177/1357633X19849280> |
| Teleconsultations reduce the transmission of COVID-19. | Monaghesh, E., & Hajizadeh, A. (2020). The role of telehealth during COVID-19 outbreak: a systematic review based on current evidence. *BMC Public Health*, *20*(1), 1193. <https://doi.org/10.1186/s12889-020-09301-4> |
| Teleconsultations are ideal for patients with long term conditions. | Ignatowicz, A., Atherton, H., Bernstein, C. J., Bryce, C., Court, R., Sturt, J., & Griffiths, F. (2019). Internet videoconferencing for patient-clinician consultations in long-term conditions: A review of reviews and applications in line with guidelines and recommendations. *Digit Health*, *5*, 2055207619845831. <https://doi.org/10.1177/2055207619845831>  Mallow, J. A., Petitte, T., Narsavage, G., Barnes, E., Theeke, E., Mallow, B. K., & Theeke, L. A. (2016). The Use of Video Conferencing for Persons with Chronic Conditions: A Systematic Review. *Ehealth Telecommun Syst Netw*, *5*(2), 39-56. <https://doi.org/10.4236/etsn.2016.52005>  Verhoeven, F., van Gemert-Pijnen, L., Dijkstra, K., Nijland, N., Seydel, E., & Steehouder, M. (2007). The contribution of teleconsultation and videoconferencing to diabetes care: a systematic literature review. *J Med Internet Res*, *9*(5), e37. <https://doi.org/10.2196/jmir.9.5.e37> |
| Teleconsultations improve access to healthcare for remote populations. | Henry, B. W., Block, D. E., Ciesla, J. R., McGowan, B. A., & Vozenilek, J. A. (2017). Clinician behaviors in telehealth care delivery: a systematic review. *Clinical Psychology Review*, *22*(4), 869-888. <https://doi.org/10.1007/s10459-016-9717-2>  Mallow, J. A., Petitte, T., Narsavage, G., Barnes, E., Theeke, E., Mallow, B. K., & Theeke, L. A. (2016). The Use of Video Conferencing for Persons with Chronic Conditions: A Systematic Review. *Ehealth Telecommun Syst Netw*, *5*(2), 39-56. <https://doi.org/10.4236/etsn.2016.52005>  Natafgi, N., Shane, D., Ullrich, F., Mackinney, A., Bell, A., & Ward, M. (2017). Using tele-emergency to avoid patient transfers in rural emergency departments: an assessment of costs and benefits. *Journal of Telemedicine and Telecare*, *24*, 1357633X1769658. https://doi.org/10.1177/1357633X17696585 |
| Collaboration and communication with other health workers is easier via videoconsultation. | Deldar, K., Bahaadinbeigy, K., & Tara, S. M. (2016). Teleconsultation and clinical decision making: a systematic review. *Acta Informatica Medica*, *24*(4), 286-292. <https://doi.org/10.5455/aim.2016.24.286-292>  Penny, R. A., Bradford, N. K., & Langbecker, D. (2017). Registered nurse and midwife experiences of using videoconferencing in practice: A systematic review of qualitative studies. *Journal of Clinical Nursing*, *27*, e739-e752. <https://doi.org/10.1111/jocn.14175> |
| Teleconsultations decrease the amount of unnecessary medical procedures. | Deldar, K., Bahaadinbeigy, K., & Tara, S. M. (2016). Teleconsultation and clinical decision making: a systematic review. *Acta Informatica Medica*, *24*(4), 286-292. <https://doi.org/10.5455/aim.2016.24.286-292>  Maarop, Nurazean; Singh, Sukdershan Singh Hazara; and Win, Khin Than: Teleconsultation technology and its benefits: In the case of public hospitals in Malaysia 2011. <https://ro.uow.edu.au/infopapers/3847> |
| Health services can be offered to underserved areas. | Mallow, J. A., Petitte, T., Narsavage, G., Barnes, E., Theeke, E., Mallow, B. K., & Theeke, L. A. (2016). The Use of Video Conferencing for Persons with Chronic Conditions: A Systematic Review. *Ehealth Telecommun Syst Netw*, *5*(2), 39-56. <https://doi.org/10.4236/etsn.2016.52005>  Natafgi, N., Shane, D., Ullrich, F., Mackinney, A., Bell, A., & Ward, M. (2017). Using tele-emergency to avoid patient transfers in rural emergency departments: an assessment of costs and benefits. *Journal of Telemedicine and Telecare*, *24*, 1357633X1769658. <https://doi.org/10.1177/1357633X17696585> |
| Patients can have teleconsultations with various health workers at the same time. | Deldar, K., Bahaadinbeigy, K., & Tara, S. M. (2016). Teleconsultation and clinical decision making: a systematic review. *Acta Informatica Medica*, *24*(4), 286-292. <https://doi.org/10.5455/aim.2016.24.286-292>  Penny, R. A., Bradford, N. K., & Langbecker, D. (2017). Registered nurse and midwife experiences of using videoconferencing in practice: A systematic review of qualitative studies. *Journal of Clinical Nursing*, *27*, e739-e752. <https://doi.org/10.1111/jocn.14175> |

| **Disadvantages** | **Supporting references** |
| --- | --- |
| Technical problems can interrupt or delay videoconsultations. | Almathami, H. K. Y., Win, K. T., & Vlahu-Gjorgievska, E. (2020). Barriers and facilitators that influence telemedicine-based, real-time, online consultation at patients' homes: systematic literature review. *J Med Internet Res*, *22*(2), e16407. <https://doi.org/10.2196/16407>  Penny, R. A., Bradford, N. K., & Langbecker, D. (2017). Registered nurse and midwife experiences of using videoconferencing in practice: A systematic review of qualitative studies. *Journal of Clinical Nursing*, *27*, e739-e752. <https://doi.org/10.1111/jocn.14175> |
| Teleconsultations are incompatible with physical examinations. | Car, J., Koh, G. C., Foong, P. S., & Wang, C. J. (2020). Video consultations in primary and specialist care during the covid-19 pandemic and beyond. *Bmj*, *371*, m3945. <https://www.doi.org/10.1136/bmj.m3945> |
| Not all health workers and/or patients have the technical resources for teleconsultations (e.g. video equipment). | Ignatowicz, A., Atherton, H., Bernstein, C. J., Bryce, C., Court, R., Sturt, J., & Griffiths, F. (2019). Internet videoconferencing for patient-clinician consultations in long-term conditions: A review of reviews and applications in line with guidelines and recommendations. *Digit Health*, *5*, 2055207619845831. <https://doi.org/10.1177/2055207619845831> |
| Not all health workers and/or patients are trained in or have the competencies for teleconsultations. | Henry, B. W., Block, D. E., Ciesla, J. R., McGowan, B. A., & Vozenilek, J. A. (2017). Clinician behaviors in telehealth care delivery: a systematic review. *Clinical Psychology Review*, *22*(4), 869-888. <https://doi.org/10.1007/s10459-016-9717-2>  Ignatowicz, A., Atherton, H., Bernstein, C. J., Bryce, C., Court, R., Sturt, J., & Griffiths, F. (2019). Internet videoconferencing for patient-clinician consultations in long-term conditions: A review of reviews and applications in line with guidelines and recommendations. *Digit Health*, *5*, 2055207619845831. <https://doi.org/10.1177/2055207619845831>  Schreiweis, B., Pobiruchin, M., Strotbaum, V., Suleder, J., Wiesner, M., & Bergh, B. (2019). Barriers and facilitators to the implementation of eHealth services: systematic literature analysis. *J Med Internet Res*, *21*(11), e14197. <https://doi.org/10.2196/14197> |
| Teleconsultations increase concerns about confidentiality and data security. | Henry, B. W., Block, D. E., Ciesla, J. R., McGowan, B. A., & Vozenilek, J. A. (2017). Clinician behaviors in telehealth care delivery: a systematic review. *Clinical Psychology Review*, *22*(4), 869-888. <https://doi.org/10.1007/s10459-016-9717-2>  Ignatowicz, A., Atherton, H., Bernstein, C. J., Bryce, C., Court, R., Sturt, J., & Griffiths, F. (2019). Internet videoconferencing for patient-clinician consultations in long-term conditions: A review of reviews and applications in line with guidelines and recommendations. *Digit Health*, *5*, 2055207619845831. <https://doi.org/10.1177/2055207619845831>  Schreiweis, B., Pobiruchin, M., Strotbaum, V., Suleder, J., Wiesner, M., & Bergh, B. (2019). Barriers and facilitators to the implementation of eHealth services: systematic literature analysis. *J Med Internet Res*, *21*(11), e14197. <https://doi.org/10.2196/14197> |
| Delivering difficult or upsetting news during teleconsultations is harder. | Car, J., Koh, G. C., Foong, P. S., & Wang, C. J. (2020). Video consultations in primary and specialist care during the covid-19 pandemic and beyond. *Bmj*, *371*, m3945. <https://www.doi.org/10.1136/bmj.m3945> |
| Limited non-verbal cues can cause communication problems and difficulties. | Henry, B. W., Block, D. E., Ciesla, J. R., McGowan, B. A., & Vozenilek, J. A. (2017). Clinician behaviors in telehealth care delivery: a systematic review. *Clinical Psychology Review*, *22*(4), 869-888. <https://doi.org/10.1007/s10459-016-9717-2> |

1: Table showing the references that informed the ranking items used in the questionnaire.
