# Supplementary material for: Medical students’ and educators’ opinions of teleconsultation in practice and undergraduate education: A UK-based mixed-methods study
Source: PLoS One. 2025 Mar 6;20(3):e0302088. doi: 10.1371/journal.pone.0302088 (PMC11884699; doi:10.1371/journal.pone.0302088)
Supplement: S3 File — This topic guide was used to guide the discussion in the semi-structured interviews. (DOCX) [file pone.0302088.s003.docx]

# S3 File

**Script for interviewer**

Thank you for agreeing to take part in this interview.

The purpose of this interview is to discuss your thoughts on TC in medical education. To reiterate the information that you have received about the study prior to giving consent, I would like to remind you that the interview will be recorded, and notes will be taken throughout. Your participation in this study will be kept confidential and all information that you provide will not contain any identifying information to link you this study. It is anticipated that this interview will last no longer than 30 minutes.

Before we begin, do you have any questions?

Do you consent for this interview to proceed?

Do you consent for this interview to be recorded?

**Opening question**

| You completed an online survey with regards to your perception of TC During the COVID-19 pandemic, face to face contact with patients had to be reduced to a minimum but it was essential to ensure an ongoing learning experience for students. | |
| --- | --- |
| Student | **Medical educators** |
| The purpose of this interview is to get insight in the way telecommunication was delivered to you and how you experienced teaching and learning during the pandemic. | Some of the question will be about your experiences as a practitioner but the main focus will be the experiences you had in facilitating students using teleconsultations and telecommunication. |
| Can you tell me a bit more about your involvement with TC. In which setting did you perform telecommunication and how did you perceive this experience? | In relation to educating students: Can you tell me a bit more about the experiences you had with facilitating students’ patient contact via TC (either in the academic setting or the clinical setting)? |
| What do you think is important to learn about teleconsultation? | What do you think is important in educating students in teleconsultation? |

**Further question**

| Student | Medical educators |
| --- | --- |
| Consider your future practice and how you think you will use teleconsultation. How will you decide whether you will use TC in your practice? | How do decide whether you use TC in your practice? |
|  | How do you decide whether you facilitate teach TC to students? |
|  | What is important for you in your practice to know about teleconsultation? What skills do you need? How prepared to you feel for doing teleconsultations? |

**Guide topics as discussion unfolds**

| Student | Medical educators |
| --- | --- |
| Influence of teleconsultation on learning outcomes and learning strategies. | Changes in delivery mode of student education regarding patient contacts. |
| Perceived advantages of this way of learning and patient contact. | Perceived advantages and facilitators of using TC in student education. |
| Perceived challenges of this way of learning and patient contact. | Perceived challenges and barriers of using TC in student education. |
|  | How do you think, your personal experience in a clinical setting influences the way you facilitate learning about telemedicine and telecommunication for students? |
| What would you do different / wish to be different in the way patient contacts via teleconsultation are done in an educational setting? | |
