# Supplementary material for: Medical students’ and educators’ opinions of teleconsultation in practice and undergraduate education: A UK-based mixed-methods study
Source: PLoS One. 2025 Mar 6;20(3):e0302088. doi: 10.1371/journal.pone.0302088 (PMC11884699; doi:10.1371/journal.pone.0302088)
Supplement: Attachment — Submitted filename: Response to Reviewers - v1.1 (1).docx [file pone.0302088.s004.docx]

**Response to the Reviewers**

Thank you to the reviewers and editor for the feedback on our manuscript. We would like to highlight our responses as follows:

**Feedback from Reviewer 2:**

1. The section “Opinions of teleconsultation practice” should be shortened and presented more concisely. In its current form, it is redundant and less suitable for a scientific paper compared to a PhD thesis.
   - Original 1614 words – reduced to ….. (to be added from the “clean” revised version)
   - We shifted parts of theme 4 to theme 3 to avoid repetition and make the information more succinct.
   - In addition, we deleted 5 quotes and moved one quote to theme 6 (under opinions of teleconsultation education)
2. The role of the PhD should be highlighted in the authors' contributions rather than in the introductory phases of the work.
   - We rephrased the sentence in the introduction, highlighting that this paper is part of a larger study: “This paper focuses on the findings related to two objectives of a larger study which aimed to investigate the factors that inform the development of teleconsultation education in undergraduate medical education in the UK.”
   - Additionally, we added a statement below the “Author contributions”: “This study was part of the first author’s PhD supervised by VOC and AOM at the School of Medicine, University of St Andrews (Scotland).”
3. Describe the age of the participants.
   - We did not collect information on the participants’ ages (neither in the questionnaire nor the interviews). We acknowledge that this might pose a limitation, however, due to the explorative nature of this study, we believe that this information would not add additional information relevant to exploring students’ and educators’ opinions of teleconsultation in medical education. We added a statement in the subsection “Study limitations” – see next comment.
4. The authors could highlight whether any sex-related differences emerged.
   - This was – similar to point 3 – not the focus of this study. We flagged this in the subsection “Study limitations”: “Due to the exploratory nature of this mixed-methods study, in-depth interferential statistics that allowed an explanatory comparison between and adjustment for participants’ characteristics such as age and gender were not prioritised. Future studies, teleconsultation education evaluations, or quality improvement projects could consider these variables in their data analysis plan.”
5. In the introduction and/or discussion, it would be helpful to add references to recent manuscripts (e.g., Health Serv Res. 2024 Aug 2;24(1):885. doi: 10.1186/s12913-024-11365-6).
   - Thank you for bringing this study from Italy to our attention. We acknowledge the fast-developing area of teleconsultation/telehealth research and added this and other references:
   - **Recommended study = reference #34:** Marsilio, M., Calcaterra, V., Infante, G., Pisarra, M., & Zuccotti, G. (2024). The digital readiness of future physicians: Nurturing the post-pandemic medical education. *BMC Health Services Research*, *24*(1), 885. <https://doi.org/10.1186/s12913-024-11365-6>
   - **Additional review = reference #21:** Alashek, W. A., & Ali, S. A. (2024). Satisfaction with telemedicine use during COVID-19 pandemic in the UK: A systematic review. *Libyan Journal of Medicine*, *19*(1), 2301829. <https://doi.org/10.1080/19932820.2024.2301829>
   - **Additional study = reference #87:** Davies, L., Lawford, B., Bennell, K. L., Russell, T., & Hinman, R. S. (2023). Telehealth education and training in entry-to-practice physiotherapy programs in Australian universities: A qualitative study with university educators. *Musculoskeletal Care*, *21*(2), 491–501. <https://doi.org/10.1002/msc.1723>
6. How were the interview questions selected?
   - Thank you for highlighting the lack of reporting here. We added the following statement to the subsection “Development and administration of the research instrument”: “The questions were informed by qualitative research published in this field and related to previous learning and practical application of teleconsultation (54) and the opinions on the implementation of teleconsultation in medical education (54, 55).”
